# Cigarette smoking and prostate cancer: A systematic review and meta-analysis of prospective cohort studies

**DOI:** 10.18332/tid/157231

**Published:** 2023-02-06

**Authors:** Sarah Al-Fayez, Ashraf El-Metwally

**Affiliations:** 1 College of Public Health and Health Informatics, King Saud Bin Abdulaziz University for Health Sciences, Riyadh, Saudi Arabia; 2 King Abdullah International Medical Research Center, Riyadh, Saudi Arabia

**Keywords:** smoking, tobacco, prostate, cancer, cigarettes

## Abstract

**INTRODUCTION:**

Cigarette smoking is a well-known cancer-causing behavior and a leading cause of death from cancer. However, according to previously published research and meta-analyses, cigarette smoking has a significant inverse association with prostate cancer incidence. Therefore, this study aims to investigate this association based on updated evidence by conducting a systematic review and meta-analysis.

**METHODS:**

A search for relevant articles was performed in PubMed and Scopus databases to obtain the pooled relative risk (RR) and the corresponding 95% confidence intervals (CIs) for the risk of prostate cancer incidence among smokers compared to non-smokers. Our search was limited to prospective cohort studies.

**RESULTS:**

A total of 17 cohort studies were included in the systematic review. Fifteen studies were included in the meta-analysis and showed that cigarette smoking has an inverse association with prostate cancer incidence with a relative risk of 0.84 (95% CI: 0.78–0.91). From all cohorts included in this systematic review, five studies examined the association between current smokers and the risk of death from prostate cancer. Therefore, a meta-analysis of these cohort studies was performed and showed that current smokers had a 42% higher risk of death from prostate cancer when compared to non-smokers with a relative risk of 1.42 (95% CI: 1.20–1.68).

**CONCLUSIONS:**

Data from observational studies suggest that cigarette smoking has an inverse association with prostate cancer incidence. However, smokers have an increased risk of death from prostate cancer. Important to realize that this lower risk for smokers might be attributed to low prostate cancer screening uptake among smokers, misclassification bias, or selection bias from the included original studies. In summary, our results indicate that the incidence of prostate cancer is lower among smokers. Nevertheless, smokers who develop the disease have a significantly worse prognosis.

## INTRODUCTION

Globally, prostate cancer (PCa) is the second leading cause of cancer and the fifth leading cause of cancer mortality among men worldwide^1^. The burden of PCa is predicted to grow by nearly around 2.3 million incidences and 740000 deaths worldwide by 2040^2^. In the USA, there were an estimated 248530 new cases and 34130 deaths from PCa in the year 2021^3^. The reported incidence of PCa in developing countries is lower than in the developed countries. It is not clear what the reason is; however, it might be due to underreporting from diagnosing centers to the national cancer registry and the geographical variation reflected by ethnic and racial dissimilarity^4,5^. Worldwide the incidence of PCa has increased remarkably, which might be attributed to the increased screening uptake among men for prostate-specific antigen (PSA) without disease symptoms^6^.

Few risk factors have been identified and linked with PCa, such as a well-connected factor of racial and ethnic variation affecting the incidence of PCa. African American and Hispanic males were more likely to be diagnosed with advanced-stage PCa compared with non-Hispanic White males. Similarly, recent research demonstrated that family history and genetic factors play a role in the risk of having PCa^7^.

Concerning age, like other types of cancer, it seems that the risk of PCa rises markedly after the age of 50 years; this might be related to the accumulated exposure to risk factors over the years. Other risk factors associated with prostate cancer but with lower evidence for a causal association, are sexually transmitted disease, unhealthy diet, inflammation, obesity, and smoking^3,8^.

Regarding smoking, burning tobacco and inhaling the smoke is considered a significant risk factor or direct cause of cancer, tumor lesions, and a well-known chemical carcinogen. Yet, it is still debatable whether cigarette smoking is a causative factor of PCa^9,10^.

Many studies have reported that smoking and prostate cancer together can lead to a higher mortality rate, especially in heavy smokers^11,12^. A meta-analysis on PCa death rates among smokers showed that smoking is a modifiable environmental risk factor leading to high death rates among PCa patients. However, smoking by itself is a low-risk factor for developing prostate cancer^13^. Similarly, De Nunzio et al.^14^, reported in their systematic review that smokers have a higher mortality rate and worse outcomes after treatment.

On the other hand, Huncharek et al.^10^ conducted a meta-analysis on 24 observational studies and found that the effect of smoking by itself on PCa incidence is weak. In fact, several cohort studies showed that smoking has a significant inverse association with PCa incidence^15-19^.

In contrast, Ho et al.^20^, in their primary research published in 2014, studied 6240 men and reported that smoking was a risk factor for developing high-grade prostate cancer^20^. Whereas Butler et al.^21^ conducted a study on 27293 Chinese and Singaporean men in 2006 and found that smoking is not considered a risk factor for developing PCa. However, the same study found a slight association between those who started smoking at an early age against those who started late.

Current epidemiological evidence shows conflicting evidence about the association between cigarette smoking and risk of PCa. To the best of our knowledge, a previous systematic review and meta-analysis conducted in 2010 showed no association between smoking and PCa^10^, while the latest in 2014 showed an inverse association between cigarette smoking and PCa^13^. Therefore, this systematic review and meta-analysis aimed to assess the association between cigarette smoking and PCa risk, based on updated cohort studies.

## METHODS

### Literature search strategy

The search of study of interest was done using MEDLINE and SCOPUS databases using keywords: smoking, tobacco, prostate, cancer, cigarettes, and tumor. Boolean operators (OR and AND) were used to reach published primary research that explicitly investigated this research question. The truncation * and quotation marks were used whenever appropriate to avoid missing any relevant articles when using our main keywords. In addition to keywords search, Mesh terms were exploded in PubMed to reach all articles relevant to our research question. Bibliographies were screened to conduct cross-referencing following the identification of relevant articles. Our search was limited to articles published in the English language between 2000 and October 2021. All through the search process, we followed PRISMA statement guidelines (preferred reporting item for systematic reviews and meta-analysis).

### Eligibility criteria

Given that it is a systematic review and meta-analysis, our study sample was relevant published articles. Cohort study designs in the human population were included in our search. Studies that assess the risk of prostate cancer in current smokers compared to non-smokers were eligible in the meta-analysis. The minimum requirement for any study included in the meta-analysis was to that the analysis adjusted for age as a confounder. We excluded published narrative or systematic reviews, meta-analysis, case reports, letters to the editor, editorials, case-control, cross-sectional, and guidelines. The inclusion criteria of articles concerning the exposure were broad, and we accepted all types of assessments of cigarette smoking. However, the inclusion criteria for the outcome were limited to studies that used biopsy as a diagnostic tool, identification through cancer registries, and formal physician diagnosis of prostate cancer. Studies that used subjective assessment methods of occurrence of prostate cancer (e.g. by self-report directly from patients or family) were excluded.

### Data extraction and quality assessment

We did not attempt to contact the authors of any of the studies to request data or any additional information. The following information was extracted from each study: the first author’s surname, publication year, location (country or region) of the conducted study, follow-up time, the total number of participants and cancer cases identified among them, age of the participants, smoking categorization used, relative risk (RR) or hazard ratio (HR) estimate for each smoking category, and the corresponding 95% confidence interval, and finally the confounder variable that was adjusted for in the analysis. Data were independently extracted by two reviewers and the quality of the included studies was assessed independently as well using the New Castle Ottawa quality assessment scale (NOS) for cohort studies. A NOS score between 0 and 9 was allocated to each study. Studies with a score of ≥7 were considered high-quality studies, while studies that scored 4–6 were considered of moderate quality, and score ≤3 of poor quality. Conflicting assessments were resolved through discussion.

### Statistical analysis

The association between cigarette smoking and prostate cancer incidence and mortality was determined based on the relative risk (RR) and the 95% confidence interval (CIs) for each study. The hazard ratio (HR) is considered equivalent to (RR) in prospective cohort studies; therefore, all risk estimates were presented as relative risk.

We calculated the pooled relative risk estimate for current smokers and the risk of prostate cancer incidence and mortality. We used a random effect model when heterogeneity was significantly high and a fixed-effect model when heterogeneity diminished. The size of heterogeneity was determined by the I^2^ statistic, Cochran’s chi-squared (Q-test), and p<0.10 indicating significant heterogeneity. The heterogeneity points out any variability in the association between smoking and prostate cancer among diverse studies. The presence of significant heterogeneity suggests that the variation between studies is not due to chance alone. The I^2^ statistic ranges from 0% to 100%. The interpretation of these values falls into two categories below and above 50%, which indicate low and high heterogeneity, respectively. A sensitivity analysis was performed by excluding studies with lower quality scores and decreased follow-up time to examine the influence of such articles on the result of the crude meta-analysis. We used funnel plots for evaluating the existence of publication bias. The funnel is a scatter plot of the number of studies included in the meta-analysis; with the value of smoking effect on the horizontal axis, and the weight of the study, such as the sample size and inverse standard error, on the vertical axis. As the sample size in the studies increases, the precision in evaluating the association between smoking and prostate cancer increases. An asymmetrical pattern in the funnel plot may indicate the presence of publication bias. Statistical analyses were conducted using RevMan software (version 5.4).

## RESULTS

### Characteristics of studies

**Figure 1:**
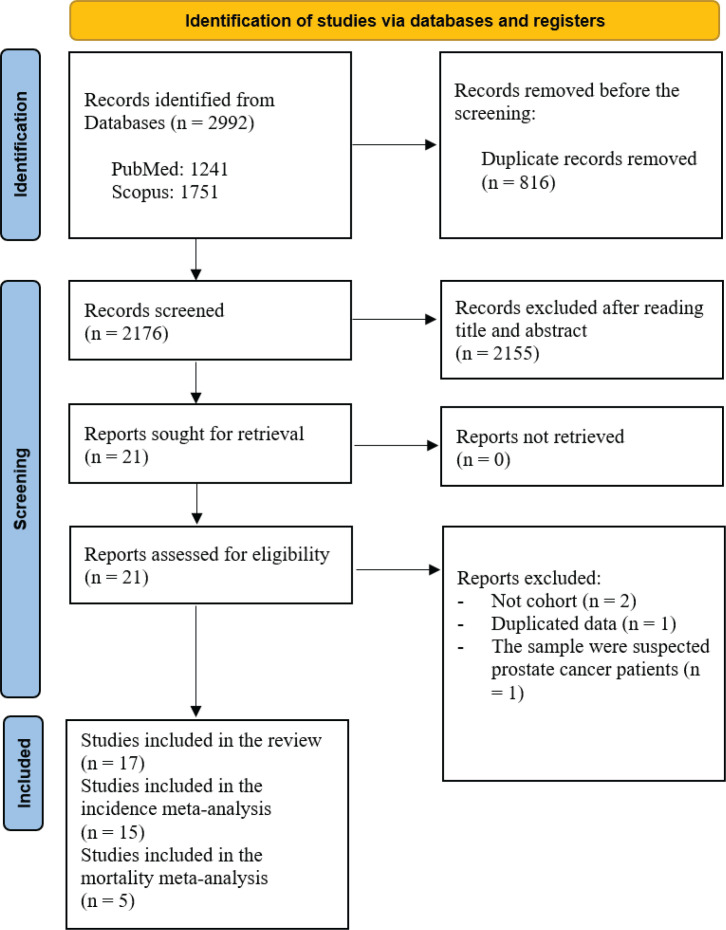
PRISMA flowchart identifying the studies that were included in the systematic review and meta-analysis

A Preferred Reporting Item for Systematic Reviews and Meta-Analysis (PRISMA) flow diagram that summarizes the search strategy is shown in Figure 1. The systematic literature search yielded a total of 2176 articles. After removing those with irrelevant title and or abstract, there were 21 relevant studies that assessed the association between cigarette smoking and the risk of prostate cancer incidence. We included 17 articles that met our inclusion and exclusion criteria in the systematic review schedule. The selected studies were published between 2000 and 2019. Finally, 15 articles were suitable for inclusion in the meta-analysis. From all cohorts included in this systematic review, five studies examined the association between current smokers and the risk of death from prostate cancer. Therefore, a fixed effect meta-analysis of these cohort studies was performed due to insignificant heterogeneity. Mostly, all cohorts were drawn from the general population except for two studies that represented selected groups of physicians and health professionals^22,23^.

A total of 2537664 participants and 47536 incident cases were included. Seven reports (42%) were from North America^11,17,18,22-25^. Approximately the same proportion of studies was carried out in the United Kingdom^16,26,27^ and European countries^19,28,29^, while the remaining four studies were conducted in Asian countries^15,21,30,31^.

All included studies in the systematic review are summarized in Table 1. Smoking status was assessed using a questionnaire in all included studies, except one study that used an in-person interview to assess smoking status^21^. Identification of smoking categories was based on a binary measure of non-smoker versus current smokers and former smokers. However, some studies have more quantitative details for smoking, such as the number of cigarettes smoked per day, age started smoking, and pack-years smoked.

**Table 1:** Main characteristics of all included studies

*Authors Year Location*	*F/U period (years)*	*Number of men (prostate cancer incidence)*	*Age (years)*	*Smoking categories*	*RR (95% CI)*	*Adjusted variables*	*(NOS)*
Nilsen et al.^28^ 2000 Europe	12	22895 (644)	≥40	Never smoker Former smoker Current smoker	(Ref.) 0.98 (0.80–1.19) 0.96 (0.78–1.19)	Age	7
Lotufo et al.^22^ 2000 United States	12.5	21985 (996)	40–84	Never smoker Former smoker Current smoker: <20 cigs/d ≥20 cigs/d	(Ref.) 1.11 (0.98–1.28) 1.04 (0.73–1.48) 1.07 (0.82–1.41)	Age, BMI, height, PI, alcohol consumption, other	6
Putnam et al.^24^ 2000 United States	6	1572 (101)	≥40	Never smoker Former smoker Current smoker: <20 cigs/d ≥20 cigs/d	(Ref.) 1.4 (0.90–2.3) 1.3 (0.60–2.8) 1.6 (0.70–3.9)	Age	6
Rohrmann et al.^11^ 2007 United States	15–19	26810 (147)	≥18	Never smoker Former smoker Current smoker ≥20 cigs/d	(Ref.) 1.33 (0.85–2.10) 1.00 (0.63–1.59) 1.38 (0.75–2.54)	Age	5
		28292 (351)		Never smoker Former smoker Current smoker ≥20 cigs/d	(Ref.) 1.04 (0.80–1.36) 0.98 (0.73–1.33) 1.01 (0.64–1.57)		
Giovannucci et al.^23^ 2007 United States	16	51529 (3544)	40–75	Never smoker Current smoker	(Ref.) 0.98 (0.89–1.07)	Age, BMI, vigorous activity, family history of prostate cancer, other	7
Butler et al.^21^ 2009 Singapore	10.4	27293 (250)	45–74	Never smoker Former smoker Current smoker	(Ref.) 1.06 (0.78–1.44) 0.88 (0.65–1.19)	Age, education, vitamin D, other	9
Watters et al.^18^ 2009 United States	NR	283112 (16640)	50–71	Never smoker Former smoker Current smoker	(Ref.) 0.90 (0.87–0.93) 0.85(0.80–0.90)	Age, race, education, marital status, PSA screening test, BMI, other	6
Rohrmann et al.^19^ 2012 Europe	Median 11.9	145112 (4623)	35–70	Never smoker Former smoker Current smoker	(Ref.) 0.96 (0.90–1.03) 0.90 (0.83–0.97)	Height, weight, education, marital status, and vigorous PI	7
Sawada et al.^30^ 2013 Japan	16	48218 (913)	NR	Never smoker Former smoker Current smoker: 0–20 20–40 ≥40 pack-years	(Ref.) 0.84 (0.70–0.99) 0.67 (0.49–0.91) 0.84 (0.70–1.02) 0.80 (0.65–0.99)	Age, public health center area, alcohol use, BMI, marital status, hx of diabetes, other	8
Bae et al.^31^ 2013 Korea	16	14450 (87)	40–59	Never smoker Former smoker Current smoker	(Ref.) 0.60 (0.34–1.06) 0.70 (0.43–1.13)	Age	6
Everatt et al.^29^ 2014 Europe	30	6976 (336)	40–59	Never smoker Former smoker Current smoker	(Ref.) 0.97 (0.74–1.26) 0.76 (0.59–1.00)	Age, education, alcohol use, and BMI	8
Park et al.^17^ 2015 United States	Mean 13.9	75216 (7115)	45–75	Never smoker Former smoker ≥20 cigs/d Current smoker ≥20 cigs/d	(Ref.) 0.84 (0.78–0.91) 0.72 (0.63–0.83)	Age, race/ethnicity, and family history for prostate cancer	7
Perez-Cornago et al.^16^ 2017 UK	5.6	219355 (4575)	40–69	Never smoker Former smoker Current smoker	(Ref.) 0.93 (0.88–0.99) 0.85 (0.77–0.95)	Age, ethnicity, lives with wife or partner, BMI, physical activity, diabetes, family history of prostate cancer, other	6
Kim et al.^15^ 2018 Korea	8	1179172 (3593)	NR	Never smoker Former smoker Current smoker	(Ref.) 0.94 (0.85–1.05) 0.77 (0.71–0.84)	Age, BMI, family history of cancer, alcohol consumption, other	6
Elwood et al.^27^ 2018 UK	Median 5.1	169715 (3220)	40–69	Current smoker Non-smoker	(Ref.) 1.11 (0.98–1.26)	Age, Townsend deprivation score, and height	4
Jacob et al.^26^ 2018 UK	30	205936 (N/A)	18–70	Non-smoker Smoker	(Ref.) 0.71 (0.65–0.76)	Age, BMI, other	8
Viner et al.^25^ 2019 Canada	Mean 12.3	10026 (401)	35–69	Never smoker Former smoker Current smoker	(Ref.) 0.85 (0.69–1.06) 0.70 (0.51–0.98)	Age, marital status, education, total household income, alcohol use, other	7

BMI: body mass index. PI: physical activity. PSA: prostate-specific antigen. NOS: New Castle Ottawa quality assessment scale. RR: relative risk.

Five cohorts contained more details and statistical analysis for the number of cigarettes smoked per day in a current smoker and the risk of prostate cancer incidence compared to a non-smoker^11,17,22,24,30^. Three studies showed a minor yet insignificant increased risk of prostate cancer in current smokers who smoke ≥20 cigarettes per day^11,22,24^. In contrast, the remaining two studies showed a significant inverse association between heavy smoking and the incidence of prostate cancer^17,30^.

Five studies adjusted age as a confounder in the association between cigarette smoking and prostate cancer^11,24,27,28,31^. In contrast, the remaining twelve studies adjusted for several confounding variables such as body mass index (BMI), height, education level, family history of prostate cancer, prostate-specific antigen test, race, marital status, medication use, and other possible confounders. The last column shows the quality score for each article included in the systematic review and meta-analysis using the Newcastle Ottawa quality assessment scale for cohort studies (NOS). The vast majority of the studies scored high quality, while the rest scored moderate quality. However, one study had a possible risk of poor quality. Consequently, the study with the risk of poor quality was removed from the meta-analysis.

### Smoking and prostate cancer incidence

**Figure 2:**
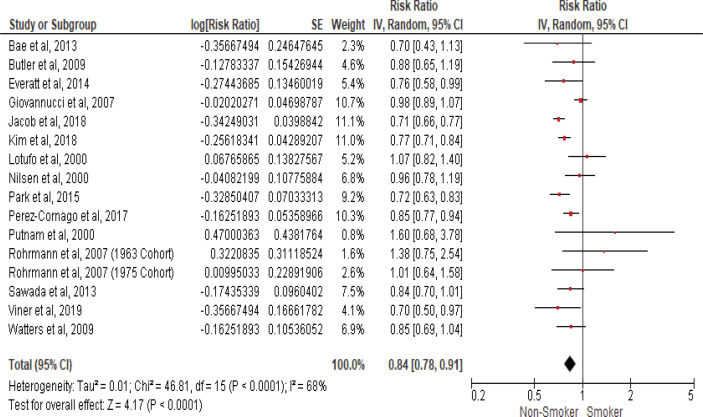
Forest plot for smoking and prostate cancer incidence

We calculated the pooled relative risk using a random effect model due to the significant heterogeny observed with I^2^ statistic (68%) and (Cochran Q) p<0.0001. The pooled estimates from fifteen cohort studies that assessed the risk of prostate cancer incidence in current smokers compared to non-smokers showed an inverse association with an RR of 0.84 (95% CI: 0.78–0.91), as shown in the forest plot (Figure 2), indicating that cigarette smoking is inversely associated with prostate cancer incidence.

### Smoking and prostate cancer mortality

**Figure 3:**
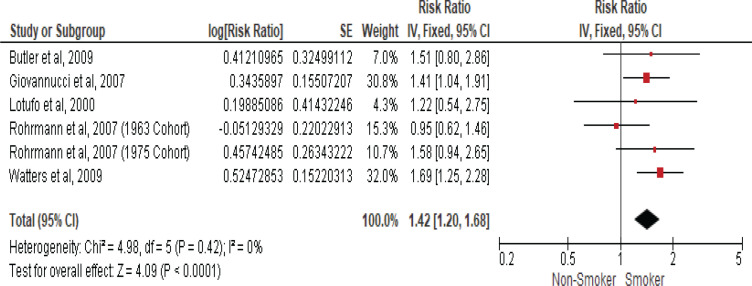
Forest plot for smoking and prostate cancer mortality

Five studies examined the association between current smokers and the risk of death from prostate cancer (Table 2). Therefore, another meta-analysis of these cohort studies was performed and showed that current smokers had a 42% higher risk of death from prostate cancer when compared to non-smokers with an RR of 1.42 (95% CI: 1.20–1.68) using a fixed-effect model due to insignificant heterogeneity with the I^2^ statistic (0%) and (Cochran Q) p<0.42 (Figure 3).

**Table 2:** Main characteristics of studies that measured mortality from prostate cancer

*Authors Year Location*	*F/U period (years)*	*Number of men (mortality from prostate cancer)*	*Age (years)*	*Smoking categorization*	*RR (95% CI)*	*Adjusted variables*	*NOS score*
Butler et al.^21^ 2009 Singapore	10.4	27293 (47)	45–74	Never smoker Recent former or current smoker	(Ref.) 1.51 (0.80–2.86)	Age, education, vitamin D, other	9
Giovannucci et al.^23^ 2007 United States	16	51529 (312)	40–75	Never smoker Current or past smoker	(Ref.) 1.41 (1.04–1.91)	Age, BMI, vigorous activity, family history of prostate cancer, other	7
Lotufo et al.^22^ 2000 United States	12.5	21985 (113)	40–84	Never smoker Former smoker Current smoker: <20 cigs/d ≥20 cigs/d	(Ref.) 1.30 (0.87–1.95) 1.25 (0.45–3.49) 1.22 (0.54–2.74)	Age, BMI, height, PI, alcohol consumption, other	6
Rohrmann et al.^11^ 2007 United States	37 years	26810 (240)	≥18	Never smoker Former smoker Current smoker ≥20 cigs/d	(Ref.) 1.01 (0.70–1.46) 0.93 (0.67–1.29) 0.95 (0.62–1.47)	Age	5
	25 years	28292 (185)		Never smoker Former smoker Current smoker ≥20 cigs/d	(Ref.) 1.02 (0.69–1.50) 1.25 (0.84–1.87) 1.58 (0.94–2.64)		
Watters et al.^18^ 2009 United States	NR	283112 (394)	50–71	Never smoker Former smoker Current smoker	(Ref.) 1.03 (0.83–1.27) 1.69 (1.25–2.27)	Age, race, education, marital status, PSA screening test, BMI, other	6

BMI: body mass index. PI: physical activity. PSA: prostate-specific antigen. NOS: New Castle Ottawa quality assessment scale. RR: relative risk.

### Publication bias

**Figure 4:**
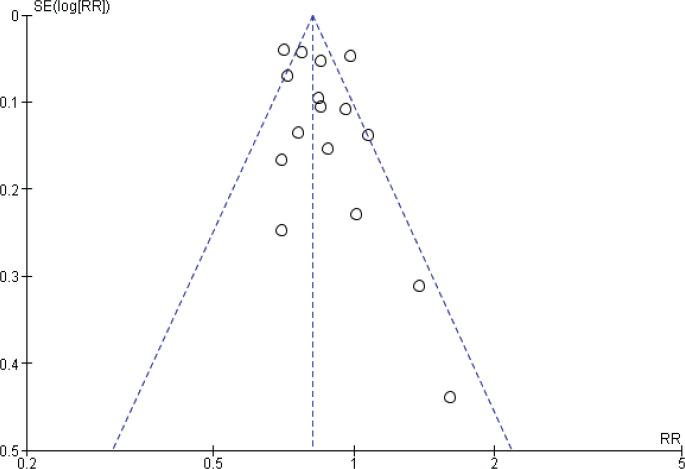
Funnel plot for incidence studies

Funnel plots were performed to assess the potential risk of publication bias. For prostate cancer incidence meta-analysis, the shape of the funnel plots might indicate a slightly asymmetrical pattern; however, this asymmetry shows two small studies as a risk factor, and our result showed inverse association, so the potential risk of publication bias is unlikely affecting our result (Figure 4).

**Figure 5:**
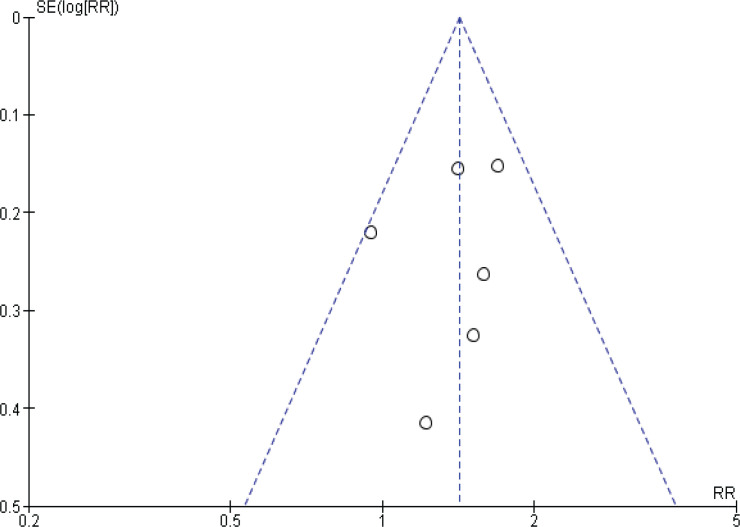
Funnel plot for mortality studies

Another funnel plot was performed to assess the risk of publication bias for prostate cancer mortality meta-analysis, the shape of the funnel plot showed no clear asymmetrical pattern, so the risk of publication bias is minimized (Figure 5).

### Statistical analysis results

A statistical analysis performed by excluding studies with (NOS) quality score equal to or below six indicating a risk of lower quality showed that smoking has inverse association with prostate cancer incidence with an RR of 0.83 (95% CI: 0.78–0.88)^11,15,16,18,22,24,31^. Similarly, another sensitivity analysis performed by excluding studies with a decreased follow-up time of 10 years or below confirmed the inverse association between smoking and the risk of prostate cancer with an RR of 0.81 (95% CI: 0.76–0.86)^15,16,21,24^. Therefore, the calculated pooled estimate in the prostate cancer incidence meta-analysis was not affected by studies with lower quality or decreased follow-up time, indicating that subgroup meta-analysis is unnecessary.

## DISCUSSION

The pooled data in the present meta-analysis enrolling more than 2500000 participants, found that cigarette smoking has a significant inverse association with prostate cancer risk. However, a meta-analysis that included studies assessing prostate cancer mortality risk among smokers showed a 42% significantly higher risk. A sensitivity analysis was conducted due to significant heterogeny in the meta-analysis that assessed the association between cigarette smoking and prostate cancer incidence; however, neither lower study quality nor decreased follow-up period exclusion changed the crude summary effect, suggesting that the observed inverse association between smoking and prostate cancer incidence is robust and reliable.

Although smoking is associated with various solid tumors^32^, the association between cigarette smoking and prostate cancer incidence seems inverse. The multifactorial etiology of prostate cancer needs to be considered when interpreting these findings. Given that most casual factors in multifactorial disease have a fairly weak effect^33^.

The result of the present meta-analysis is consistent with the previous systematic review and meta-analysis and most epidemiological observational studies that assess the risk of prostate cancer in smokers compared to non-smokers^10,13,21,26,30^. The observed inverse association between smoking and prostate cancer incidence was modest. However, the association between cigarette smoking and increased mortality from prostate cancer was robust, suggesting that smoking is associated with worse prostate cancer outcomes.

The search about prostate cancer mortality among smokers was not done comprehensively as this article focuses on the incidence of prostate cancer, and the data about mortality were only among the studies that measured incidence. The result was added to have a broader picture of the effect of smoking on prostate cancer. Notably, the result found in our meta-analysis complies with previous epidemiological evidence showing that smokers have higher prostate cancer mortality compared to non-smokers^34^.

### Strengths and limitations

This meta-analysis has several methodological strengths, including that two independent reviewers conducted the search, data extraction, and quality assessment following the PRISMA statement guidelines. Furthermore, our inclusion criteria for eligible study design were limited to cohort; therefore, the possibility of recall bias was minimized, and the clarity in temporality sequence between exposure and outcome was ascertained.

In addition, the association between cigarette smoking and mortality from prostate cancer showed no heterogeneity, indicating that the risk reported in different studies was significantly consistent. We also conducted various sensitivity analyses to examine the relationship between smoking and prostate cancer in different settings.

Furthermore, the funnel plots of incidence studies showed an asymmetrical pattern. Still, they were not indicative of publication bias, as small significant results showed smoking as a risk factor instead of being inversely associated with prostate cancer, as the summary estimate showed.

This study has a few limitations related to the included articles that might explain the inverse association observed; most of the limitations are methodological issues related to the design or data collection tools, particularly in the assessment of smoking which might not be done comprehensively. Most studies used a smoking assessment tool at the beginning of the study with three categorizations of the exposure: none, former and current smoker. Lacking repeated exposure assessment in all of the included studies might introduce the possibility of measurement bias. Then again, this limitation will increase the risk of non-differential misclassification as the exposure might change for smokers and non-smokers equally. After all, future studies need to evaluate the exposure longitudinally and assess the intensity, duration, and intermittence of smoking to reduce the possibility of measurement bias and increase the accuracy in estimating the changing spectrum of cigarette smoking habits.

Moreover, the results from most of the included articles were not presented comprehensively, as the data separating high-risk prostate cancer from low-risk prostate cancer were not mentioned in the retrieved studies^11,15-17,24-29,31^.

Additionally, none of the included studies mentioned any information or adjustment for prostate cancer screening. Ignorance of such an important confounder might lead to methodological bias in these studies. Important to realize that increasing evidence suggests smokers have lower compliance with prostate cancer screening programs and are more likely to have a high-grade disease at surgery, therefore a higher risk of metastasis, recurrence, and death^35-37^. This may be related to a delayed diagnosis among smokers or a stronger causal association of cigarette smoking with the prognosis of prostate cancer, which may be a plausible explanation for our findings. In either case, future studies need to consider the importance of adjusting for adherence to prostate cancer screening as a confounder.

Another possible explanation is that the participants who were categorized as non-smokers might be using other forms of smoking such as electronic cigarettes, hookah, or chewing tobacco, which are widely common forms of smoking. Therefore, lacking this information might explain the increased risk of prostate cancer in the non-smoker group as these details were not measured in the included articles. In order to avoid this limitation, future studies should exclude all forms of smoking in the non-smoker group when assessing the association between cigarette smoking and prostate cancer risk.

In summary, since prostate cancer is the second leading cause of cancer and the fifth leading cause of cancer mortality among men worldwide^1^, raising global awareness about the importance of increasing smoking cessation efforts may considerably reduce prostate cancer mortality.

## CONCLUSIONS

Data from observational studies suggest that cigarette smoking has an inverse association with prostate cancer incidence. However, smokers have an increased risk of death from prostate cancer. It is important to realize that this lower risk for smokers might be attributed to low prostate cancer screening uptake among smokers, misclassification bias, or selection bias from the included original studies. In summary, this result indicates that prostate cancer incidence is less among smokers, while those who smoke and develop the disease will have a significantly worse prognosis and higher mortality risk.

## Data Availability

The data supporting this research are available from the authors on reasonable request.

## References

[1] International Agency for Research on Cancer Global Cancer Observatory.

[2] Culp MB, Soerjomataram I, Efstathiou JA, Bray F, Jemal A (2020). Recent Global Patterns in Prostate Cancer Incidence and Mortality Rates. Eur Urol.

[3] American Cancer Society https://www.cancer.org/cancer/prostate-cancer.html.

[4] Sloan FA, Gelband H, Institute of Medicine (US) Committee on Cancer Control in Low- and Middle-Income Countries (2007). Cancer Control Opportunities in Low- and Middle-Income Countries.

[5] Arafa MA, Rabah DM (2017). With increasing trends of prostate cancer in the Saudi Arabia and Arab World: Should we start screening programs?. World J Clin Oncol.

[6] Bray F, Ferlay J, Soerjomataram I, Siegel RL, Torre LA, Jemal A (2018). Global cancer statistics 2018: GLOBOCAN estimates of incidence and mortality worldwide for 36 cancers in 185 countries. CA Cancer J Clin.

[7] Bratt O (2002). Hereditary Prostate Cancer: Clinical Aspects. J Urol.

[8] Gómez-Acebo I, Dierssen-Sotos T, Fernandez-Navarro P (2017). Risk Model for Prostate Cancer Using Environmental and Genetic Factors in the Spanish Multi-Case-Control (MCC) Study. Sci Rep.

[9] Secretan B, Straif K, Baan R (2009). A review of human carcinogens—Part E: tobacco, areca nut, alcohol, coal smoke, and salted fi sh. Lancet Oncol.

[10] Huncharek M, Haddock KS, Reid R, Kupelnick B (2010). Smoking as a Risk Factor for Prostate Cancer: A Meta-Analysis of 24 Prospective Cohort Studies. Am J Public Health.

[11] Rohrmann S, Genkinger JM, Burke A (2007). Smoking and risk of fatal prostate cancer in a prospective U.S. study. Urology.

[12] Kenfield SA, Stampfer MJ, Chan JM, Giovannucci E (2011). Smoking and Prostate Cancer Survival and Recurrence. JAMA.

[13] Islami F, Moreira DM, Boffetta P, Freedland SJ (2014). A Systematic Review and Meta-analysis of Tobacco Use and Prostate Cancer Mortality and Incidence in Prospective Cohort Studies. Eur Urol.

[14] De Nunzio C, Andriole GL, Thompson IM, Freedland SJ (2015). Smoking and Prostate Cancer: A Systematic Review. Eur Urol Focus.

[15] Kim SH, Kim S, Joung JY (2018). Lifestyle Risk Prediction Model for Prostate Cancer in a Korean Population. Cancer Res Treat.

[16] Perez-Cornago A, Key TJ, Allen NE (2017). Prospective investigation of risk factors for prostate cancer in the UK Biobank cohort study. Br J Cancer.

[17] Park SY, Haiman CA, Cheng I (2015). Racial/ethnic differences in lifestyle-related factors and prostate cancer risk: the Multiethnic Cohort Study. Cancer Causes Control.

[18] Watters JL, Park Y, Hollenbeck A, Schatzkin A, Albanes D (2009). Cigarette Smoking and Prostate Cancer in a Prospective US Cohort Study. Cancer Epidemiol Biomarkers Prev.

[19] Rohrmann S, Linseisen J, Allen N (2013). Smoking and the risk of prostate cancer in the European Prospective Investigation into Cancer and Nutrition. Br J Cancer.

[20] Ho T, Howard LE, Vidal AC (2014). Smoking and risk of low- and high-grade prostate cancer: results from the REDUCE study. Clin Cancer Res.

[21] Butler LM, Wang R, Wong AS, Koh WP, Yu MC (2009). Cigarette smoking and risk of prostate cancer among Singapore Chinese. Cancer Causes Control.

[22] Lotufo PA, Lee IM, Ajani UA, Hennekens CH, Manson JE (2000). Cigarette smoking and risk of prostate cancer in the physicians’ health study (United States). Int J Cancer.

[23] Giovannucci E, Liu Y, Platz EA, Stampfer MJ, Willett WC (2007). Risk factors for prostate cancer incidence and progression in the health professionals follow-up study. Int J Cancer.

[24] Putnam SD, Cerhan JR, Parker AS (2000). Lifestyle and Anthropometric Risk Factors for Prostate Cancer in a Cohort of Iowa Men. Ann Epidemiol.

[25] Viner B, Barberio AM, Haig TR, Friedenreich CM, Brenner DR (2019). The individual and combined effects of alcohol consumption and cigarette smoking on site-specific cancer risk in a prospective cohort of 26,607 adults: results from Alberta’s Tomorrow Project. Cancer Causes Control.

[26] Jacob L, Freyn M, Kalder M, Dinas K, Kostev K (2018). Impact of tobacco smoking on the risk of developing 25 different cancers in the UK: a retrospective study of 422,010 patients followed for up to 30 years. Oncotarget.

[27] Elwood PC, Whitmarsh A, Gallacher J (2018). Healthy living and cancer: evidence from UK Biobank. Ecancermedicalscience.

[28] Lund Nilsen TI, Johnsen R, Vatten LJ (2000). Socio-economic and lifestyle factors associated with the risk of prostate cancer. Br J Cancer.

[29] Everatt R, Kuzmickienė I, Virvičiūtė D, Tamošiūnas A (2014). Cigarette smoking, educational level and total and site-specific cancer: a cohort study in men in Lithuania. Eur J Cancer Prev.

[30] Sawada N, Inoue M, Iwasaki M (2014). Alcohol and smoking and subsequent risk of prostate cancer in Japanese men: The Japan Public Health Center-based prospective study. Int J Cancer.

[31] Bae JM, Li ZM, Shin MH, Kim DH, Lee MS, Ahn YO (2013). Cigarette Smoking and Prostate Cancer Risk: Negative Results of the Seoul Male Cancer Cohort Study. Asian Pac J Cancer Prev.

[32] Smoking and Cancer Centers for Disease Control and Prevention.

[33] Buchanan AV, Weiss KM, Fullerton SM (2006). Dissecting complex disease: the quest for the Philosopher’s Stone?. Int J Epidemiol.

[34] Darcey E, Boyle T (2018). Tobacco smoking and survival after a prostate cancer diagnosis: A systematic review and meta-analysis. Cancer Treat Rev.

[35] Roberts WW, Platz EA, Walsh PC (2003). Association of Cigarette Smoking with Extraprostatic Prostate Cancer in Young Men. J Urol.

[36] Moreira DM, Aronson WJ, Terris MK (2014). Cigarette Smoking Is Associated With an Increased Risk of Biochemical Disease Recurrence, Metastasis, Castration-Resistant Prostate Cancer, and Mortality After Radical Prostatectomy: Results From the SEARCH Database. Cancer.

[37] Byrne MM, Davila EP, Zhao W (2010). Cancer screening behaviors among smokers and non-smokers. Cancer Epidemiol.

